# Culturally Based Practice in Neonatal Procedural Pain Management: A Mini Review

**DOI:** 10.3389/fped.2020.00540

**Published:** 2020-09-03

**Authors:** Siti Yuyun Rahayu Fitri, Viera Wardhani, Windy Rakhmawati, Tuti Pahria, Sri Hendrawati

**Affiliations:** ^1^Pediatric nursing department, Faculty of Nursing, Universitas Padjadjaran, Bandung, Indonesia; ^2^Faculty of Medicine, Universitas Brawijaya, Malang, Indonesia; ^3^Medical Surgical Nursing Department, Faculty of Nursing, Universitas Padjadjaran, Bandung, Indonesia

**Keywords:** acupuncture, aromatherapy, culture, foot massage, neonates, pain management, procedural pain

## Abstract

Cultural factors have gradually become important considerations in health services, including in pain management for adult and pediatric patients. However, research on culturally based pain management in neonates remains limited. This mini review aims to provide an overview of procedural pain management in neonates based on cultural approaches from various countries. The review found that there are several analyses of pain management procedures for neonates developed from cultural practices, namely, acupuncture, foot massage and reflexology, Yakson touch therapy, and aromatherapy. The acupuncture method (invasive and non-invasive) is more widely studied using randomized controlled trials (RCTs) than the other methods because the techniques applied can be standardized and measured. There are indications of the positive impact of all the methods examined in this review, but the results of studies have not been consistent because of the diversity of outcome measurement methods used and because of the difficulty of creating standardized procedures to measure pain management methods that are based on cultural practices.

## Introduction

Neonates in neonatal intensive care units (NICUs) experience painful procedures, with a range between 7.5 and 17.3 per neonate per day (1); the most common procedures being heel lancing, suctioning, tape removal, nasogastric/orogastric tube insertion, intravenous line insertion, venipuncture, and peripherally inserted central catheter line insertion (2).

Prevention and treatment of pain in neonates are highly important given the impact of short-term and long-term pain on various aspects of neonatal well-being and development. Despite the many treatments that cause pain in neonates, pain management for these patients conducted by health workers remains limited (3–5). Previous studies have referred to various types of procedural pain management methods in neonates (6–11). Generally non-pharmacological treatments for pain relief in neonates can be categorized into three types of approaches: environmental control, feeding methods, and other interventions (6). We summarize the current evidence for non-pharmacological culturally based treatment for pain relief in neonates (Table 1).

**Table 1:** Non-pharmacological analgesia for procedural pain in neonates.

**Approach**	**Type of analgesia**
Environmental control	Skin to skin contact
	Kangaroo mother care
	Swaddling
	Facilitated tucking
	Therapeutic touch/massage
	Musical therapy
	Auditory recognition
	Olfactory/aromatherapy recognition
Feeding methods	Breast milk
	Breastfeeding
	Nonnutritive sucking
Other intervention	Acupuncture
	Sucrose/glucose solution
	Sensorial saturation/multisensory stimulation

Currently, cultural approaches are being increasingly applied in healthcare for neonates, including in procedural pain management. Medical professionals need to consider cultural practices that are trusted by the public when providing health services, so that such practices become more widely accepted in the community (12). In addition, culture has been found to be one of the main elements in nursing (known as cultural nursing) that makes nursing services safer and more humane (13).

Culture is a framework that guides human behavior in many situations. This means that culture can direct people in finding ways to overcome the health problems they face, including pain management. Cultural factors have driven the development of a variety of pain management methods in different community groups (14). Various studies have noted that culture influences the meaning of, and responses to, individual pain, including in children. However, most studies of cultural practices in pain management in children do not consider neonates. Sujatha (15) stated that there is a strong relationship between cultural practices in the community and neonatal care. The purpose of this mini review is to summarize the findings of studies on procedural pain management methods for neonates that are based on culture, tradition, and ethnicity. The results of the review are expected to provide an overview of the application of culturally based pain management to neonates and encourage further study to ensure that pain management procedures in neonates are safe and tested according to each culture.

## Method

A mini review was conducted to explore information on traditional or culturally based neonatal pain management in various ethnic communities. The literature search used neonatal procedural pain management as the research topic. The keywords and phrases included were a combination of “neonatal” and the search terms “culture,” “traditional,” “ethnicity,” “procedural pain,” “pain management,” “acupuncture,” “Yakson,” “massage,” “aromatherapy.” These were further enhanced using the terms “and” and “or.” The search for literature was conducted using the Scopus, Science Direct, and PubMed databases from 2000 to 2020, and restricted to English-language articles. The studies included in the review were not limited to any specific research designs.

## Results

### Acupuncture

Acupuncture is a form of culturally based pain management that has been widely studied. Acupuncture is a method of traditional Chinese medicine that has its own system for the diagnosis and treatment of disease. Scientific research into the mechanisms of acupuncture began in ~1950 when a research group at Peking University published an important pharmacological study (16). Since the 1970s, the mechanism of acupuncture analgesia has been widely explored (17). Acupuncture is based on the idea of energy (“Chi”) circulating through the body by using meridians as pathways (18). Acupuncture has begun to be applied in children, although the research on the efficacy of this method remains limited. Reviews have reported that the limitations of acupuncture application in children are caused by children's fear of needles, infants' or toddlers' lack of cooperation, risk of infection, and the lack of clarify about the safety of acupuncture given that responses to therapy are difficult to evaluate during in infancy (19).

There are two different strategies for performing acupuncture therapy: manual and electro acupuncture (17). Electro acupuncture is a modified form of manual acupuncture. Manual acupuncture or traditional acupuncture is an invasive method that involves insertion and manipulation of fine needles in specific cutaneous areas that are associated with high electrical activity and peripheral nerve connections. Recently, electro acupuncture has also begun to be used. This procedure involves using modifications of needles in acupuncture to make the procedure non-invasive, for example, magnets, electrical current without needles, lasers, pressure (acupressure), and heat (moxibustion) (20).

The use of invasive and non-invasive methods of acupuncture in neonates to reduce procedural pain has been examined only in several studies. In a study using invasive acupuncture to reduce pain in 10 neonates who had undergone the heel prick procedure, acupuncture was performed by competent doctors by inserting thin, sterile disposable needles (0.22 × 1.5 mm) at the Yintang acupuncture point (EXHN3). This acupuncture point is located midway between the medial ends of the eyebrows. Each needle was kept in place for 30 min and then removed (21). Pain was assessed using the Neonatal Infant Pain Scale (NIPS). The results showed that acupuncture using this method significantly reduced pain scores. Other than this study, no other study on invasive acupuncture using needle is available so far.

Non-invasive acupuncture has also been used in neonates undergoing hospital treatment (Table 2). This mini review identified seven research articles that examined non-invasive acupuncture in neonates (six articles on pain management caused by heel prick and one article that did not examine any specific procedure). Review of the studies found variations in the techniques and the acupuncture points used. Four non-invasive acupuncture methods were examined in the studies: acupressure, laser acupuncture, magnetic electro puncture, and electrical stimulation. Nine acupuncture points used are in the body area and five are in the auricular area. The subjective measurement tools were the NIPS and crying duration, and the objective measurement tools used were the premature infant pain profile (PIPP), heart rate variability (HRV), and saliva testing. While some of the studies reviewed found that acupuncture decreases pain scores in the objective measures, the results were not consistent across the studies.

**Table 2:** Application of noninvasive acupuncture in neonates.

**References**	**Procedure**	**Electro Acupuncture**	**Acupuncture Point**	**Patients (n)**	**Outcomes**	**Findings**	**Design**
Abbasoglu et al. (22)	Heel prick	Acupressure	BL60 (in a depression between the tip of the lateral malleolus and the Achilles tendon) K3 (medial aspect of the foot, posterior to the medial malleolus and in the depression between the tip ofthe medial malleolus and the Achilles tendon)	32	PIPP score Duration of procedure Duration of crying	No significant finding in PIPP score Significant reduction in mean duration of procedure Significant finding in mean duration of crying	Prospective RCT
Abbasoglu et al. (23)	Heel prick	Laser acupuncture (LA)	Yintang (On the forehead, at the midpoint between the two medial ends of the eyebrow)	42	NIPS Duration of procedure Duration of crying	No significant finding in NIPS score Significant reduction in mean duration of procedure Mean time in duration of crying was longer in LA	Prospective Unblind Uncontrol
Mitchell et al. (24)	Heel prick	Electrical stimulation	ST36 (one finger width lateral from the anterior crest of the tibia, in the tibialis anterior muscle) SP6 (above the tip of the medial malleolus on the posterior border of the tibia) BL60 KI3 (lower leg)	162	PIPP score HRV Salivary cortisol	No significant finding for PIPP score No significant finding for HRV No significant finding for salivary cortisol	2 x 2 factorial RCT Double blind
Yates et al. (25)	Heel prick	Electrical stimulation (1.0 mA, 2 Hz)	ST36, SP6, KI3, BL60	30	Skin assessment Vital signs PIPP score	No adverse effects No significant change in vital signs No significant change in PIPP score	Prospective Unblind Uncontrolled Open label trial
Koç Özkan et al. (26)	Heel prick	Acupressure Foot massage	UB60 (side of the ankle) K3 (side of the ankle)	139	NIPS score HR SaO_2_ Duration of crying	Significant decrease in NIPS score	RCT
Tugcu et al. (27)	–	Acupressure	Yintang (EX-HN3)	17	Neonatal skin perfusion SaO_2_ Pulse rate	Increases in peripheral perfusion Increases in SaO_2_, values Significant decrease in pulse rate	Prospective Unblind Uncontrolled
Chen et al. (28)	Heel prick	Magnetic acupuncture	Five auricular points (cingulate gyrus, thalamus, omega, point zero, and shenmen)	40	PIPP score	Significant decrease in PIPP score	RCT

*HRV, heart rate variability; PIPP, premature infant pain profile; NIPS, Neonatal Infant Pain Scale; LA, laser acupuncture; HR, heart rate; SaO_2_, oxygen saturation; RCT, randomized controlled trial*.

It remains unclear whether invasive or non-invasive acupuncture methods are more effective as analgesic interventions for pain caused by procedural treatments in neonates (19). This lack of clarity is partly because the efficacy of acupuncture depends significantly on the ability of the clinician and on the compliance of the clinician in applying principles of safety and hygiene (19, 29). However, non-invasive techniques may be more suitable for neonates given that there is no clear evidence of the short-term or long-term effects of acupuncture (20). Moreover, the skin of premature babies and newborns is very thin and shows physiologic and histologic peculiarities. This means it is important for to ensure the skin remains undamaged and that no potential entry point for infectious diseases is created (30). Thus, non-invasive interventions should be considered for premature babies and newborns (25, 31, 32). However, there are no data or research studies that prove which non-invasive methods of acupuncture are superior or which acupuncture points are safe and appropriate for neonates.

Acupuncture has the potential to decrease neonatal exposure to potentially neurotoxic analgesics and sedative agents (19). The limited data available suggest that acupuncture is a safe non-pharmacologic treatment option for pain reduction in term and preterm infants. Chen et al. (20) noted that currently, there is a need for a high-quality well-designed and standardized RCT that uses physiological outcomes to evaluate the efficacy of acupuncture. A 2016 review of acupuncture (33) concluded that studies that showed a lack of efficacy or uncertain results outweighed studies with positive results. The possible reasons for this were related to the overall quality of research, use of poorly designed sham acupuncture, lack of standard procedures for locating points and needling, and lack of attention to individual differences. Acupuncture may have a positive pain-relieving effect in neonates. However, given the low number of available high-quality trials and the lack of heterogeneity across studies, it is not possible to state clear recommendations (33, 34).

### Foot Massage and Reflexology

The most ancient references to the use of massage came from China (~2700 BC). Massage therapy can be applied to adults and children (35). Given the worldwide popularity of massage therapy, there are many common types of massage therapy available, including Swedish massage, Shiatsu, Rolfing, reflexology, myofascial release, and craniosacral therapy (36). In India, Bangladesh, Nepal, and other neighboring countries, neonatal massage has been a traditional practice (37). Massaging the feet and hands stimulates the mechanoreceptors that activate the “nonpainful” nerve fibers that prevent pain transmission from reaching consciousness (38).

Foot massage has been shown to improve physiological and behavioral stability and to lower the severity of pain responses to heel lancing in preterm infants, as well as to decrease the heel lancing duration (39). In addition, foot massage has been found to reduce pain responses to procedures other than heel stick in NICUs (40). Foot massage is considered to be as effective as acupressure in reducing pain in neonates undergoing heel stick (26).

Foot massage is often identified with foot reflexology (40, 41). However, there is a slight difference because foot reflexology is not simply foot massage (42), with the principal difference being the intensity level of the massage. A foot massage does not usually affect the deeper tissue, nor it is centered on specific pressure points. In contrast, reflexology is the study of how one part of the human body (in this case, the feet and hands) relates to other parts of the body (43) and considers that the hands and feet contain reflex zones. In addition, Foot reflexology reduces the pain in vaccinating infant aged 0–12 months (44).

Neonatal foot massage and reflexology were considered together in this review because reflexology has received very little research attention particularly in relation to procedural pain relief in neonates. The absence of standardized studies of reflexology, the inadequate use of study protocols and guidelines, and the data heterogeneity mean that the results of studies on reflexology cannot be generalized and there can be no conclusion about whether this type of massage has a positive effect on neonates in relation to procedural pain management (43). To provide any evidence for the efficacy of this method, well-structured double-blind RCTs are required.

### Yakson Touch

In Korea, Yakson culture has been explored for use as a non-pharmacological analgesic in neonates (45–50). The compound word “Yakson” comes from yak, which means medicine, and son, which means hands, thus the literal meaning of Yakson is “medicine hands” (51). Yakson touch is a traditional touching method where mothers and grandmothers caress and massage their sick children's area of pain using supernatural power (46). Yakson touch employs a pure and natural action where a mother or a grandmother puts her hand on the pain area of the child and caresses or massages the area to relieve the child's pain or discomfort. The attributes of Yakson touch are earnest wish, soothing, physical expression, mutual interaction, and family ties (51).

Several studies have examined Yakson touch as a non-pharmacologic analgesic method in neonates. Some studies apply Yakson touch to provide a calming effect on neonates and infants. Although Yakson touch is not specifically applied to reduce pain, the effect of inducing tranquility is also the basis of several non-pharmacological analgesic methods. Therefore, this review discusses Yakson touch as a method of pain relief for neonates.

Yakson touch was performed on neonates during heel stick procedures to reduce pain by maintaining oxygen saturation during the procedure, with findings of a significant effect on pain reduction (46). In the study, Yakson touch was conducted by nurses. The study also noted that Yakson touch can be performed alone or in combination with other pacifiers during the heel stick procedure.

Compared with other touch therapies, Yakson touch shows a more rapid calming effect because it stimulates a sense of comfort during the process. However, in gentle human touch (GHT) therapy, the calming effect appears only after the therapy process is completed (47). In the study, that babies who received Yakson intervention had longer sleep times. Other studies also found that Yakson touch led to high sleep scores compared with the control group (48) and the GHT group (49). As is widely acknowledged, prolonged sleep is essential for optimum healing (52). Thus, there is some evidence for the efficacy of Yakson touch as a non-pharmacological intervention for managing procedural pain in neonates.

### Aromatherapy

Aromatherapy refers to the inhalation and topical application of authentic essential oils from aromatic plants to restore or enhance health, beauty, and well-being (53). It is well known that aromatic oils were used in China, in Australia by Indigenous Australians, in India, and in ancient Egypt (53). Currently, the use of aromatherapy has been applied in healthcare, including in pediatric patients. In the field of neonatology, aromatherapy has begun to be applied to manage procedural pain. Some of the aromas used are lavender, vanilla, and breastmilk.

The aroma of breastmilk was found to have an analgesic effect on premature babies and can be used as a safe method of pain reduction (54). The aroma of breastmilk can also improve pulse and oxygen saturation when neonates undergo venipuncture procedures (55), and is used to calm premature babies during and after venipuncture (56). Another study found that breastmilk aroma had a more significant effect on changes in heart rate and oxygen saturation in neonates during and after venipuncture than the aroma of vanilla (57). It was also found to decrease heart rate variability and oxygen saturation of premature babies. In contrast, vanilla aroma was not found to create any significant changes in heart rate and oxygen saturation in neonates (57). Other studies have shown that the aroma of breastmilk and lavender can prevent increasing pulse rate and reduce oxygen saturation and pain score measured using NIPS (58). However, other studies suggest that the use of aromatherapy does not significantly affect pain (59). Despite the promising results presented here, research on the use of aromatherapy in procedural pain management in neonates remains limited and involves limited samples. Further standardized clinical trials must be conducted as well as standardizing the intervention protocols for administering aromatherapies to neonates.

## Conclusion

This mini review demonstrates the potential benefits of cultural practices in managing procedural pain in neonates despite the studies presented having limitations in their trial designs, standardization of procedures, and outcome measures. The benefits of cultural practices in pain management in neonates exist not only through the effect that can be objectively measured but also through the processes and beliefs that underlie these practices. A balanced research approach is needed to reveal the objective pain relief benefits of culturally based methods of procedural pain management in neonates and the effect of the subjective meaning of cultural approaches in such pain management.

## Author Contributions

SF: conceived and planned the review. VW: designed the study, interpreting the results and worked on the manuscript. WR: wrote the manuscript with input from all authors. TP: contributed to the interpretation of the results. SH: worked out almost all of the technical details. All authors contributed to the article and approved the submitted version.

## Conflict of Interest

The authors declare that the research was conducted in the absence of any commercial or financial relationships that could be construed as a potential conflict of interest.
